# Supplementary material for: Missense Variants Reveal Functional Insights Into the Human ARID Family of Gene Regulators
Source: J Mol Biol. 2022 May 15;434(9):167529. doi: 10.1016/j.jmb.2022.167529 (PMC9077328; doi:10.1016/j.jmb.2022.167529)

Supplementary figures for:

Missense variants reveal functional insights into the human ARID family of gene regulators

Gauri Deák and Atlanta G. Cook*
Wellcome Centre for Cell Biology, University of Edinburgh, Michael Swann Building, Max Born Crescent, Edinburgh EH9 3BF, United Kingdom

Figure S1: Modelled structure of the ARID2 equivalent of the ARID1A/1B CBRB (annotated with missense variants **(A)** and sequence conservation in metazoa **(B)**

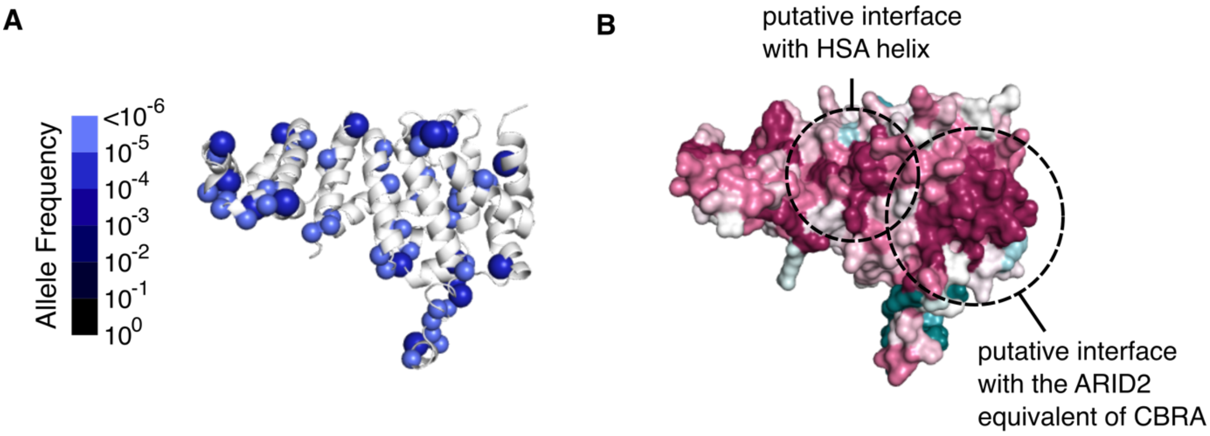

Figure S2: Solved structure of JARID1B (5FUP) annotated with missense variants **(A)** and sequence conservation in metazoa **(B)**

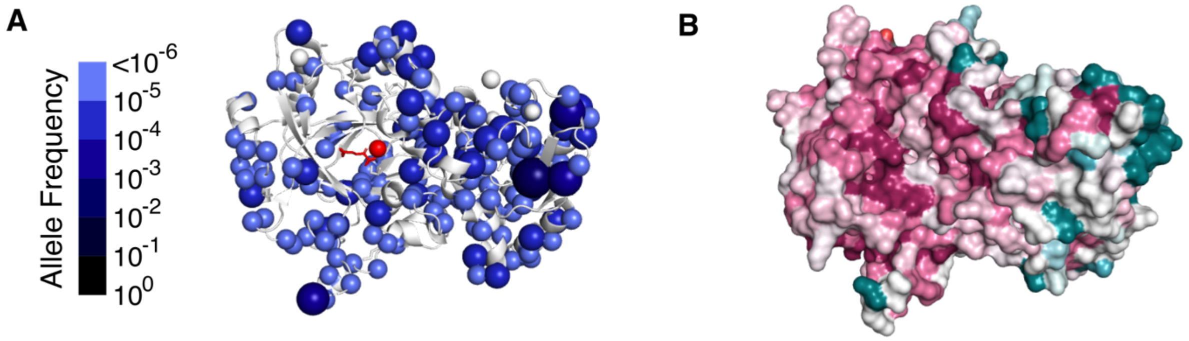

Figure S3: 1D plots of missense variants in ARID4A **(A)** and ARID4B **(B)** and the V_d/V_p ratios calculated for their functional domains

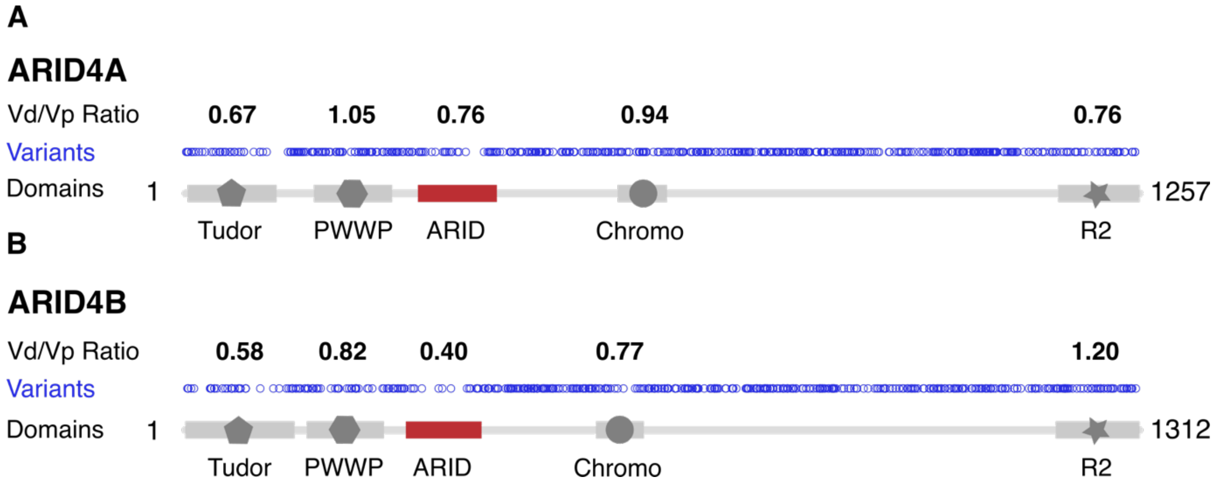

Figure S4: 1D plots of missense variants in ARID3A **(A)**, ARID3B **(B)**, and ARID3C **(C)** and the V_d/V_p ratios calculated for their functional domains

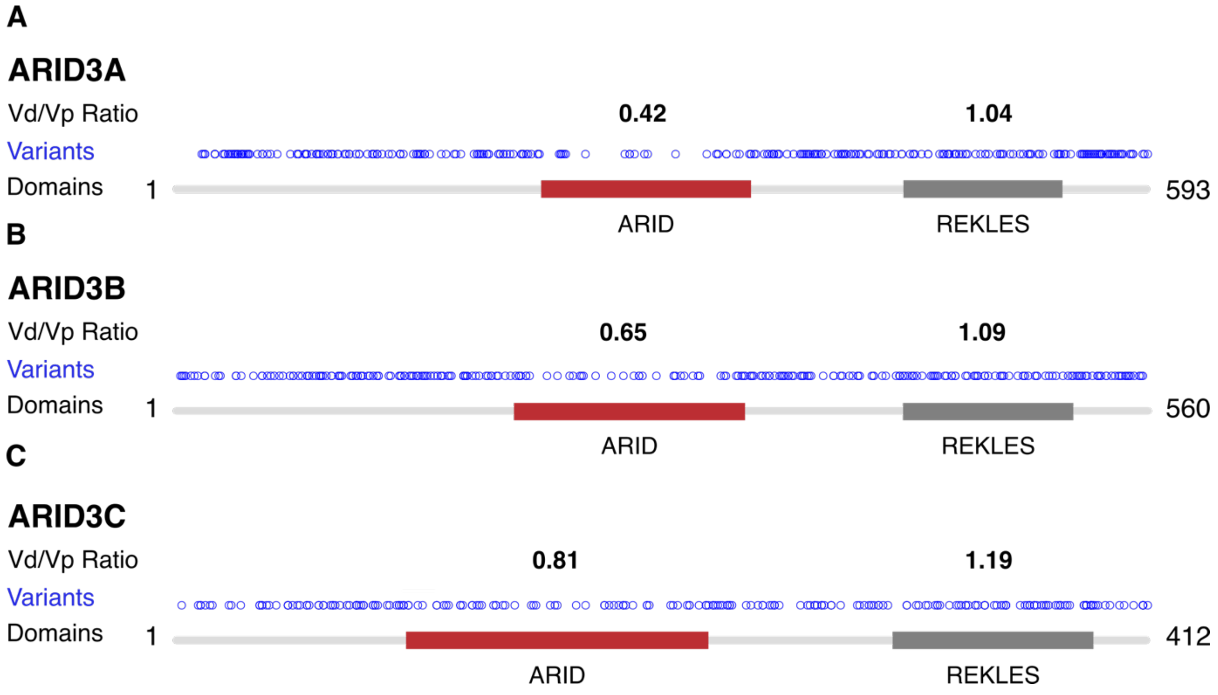

Figure S5: A vertebrate multiple sequence alignment of the N-terminal region of ARID5B isoform 1; The end of the BAH domain and the start of the C-terminal extension is marked by a vertical line at human aa 107. Darker blue represents higher percentage identity.

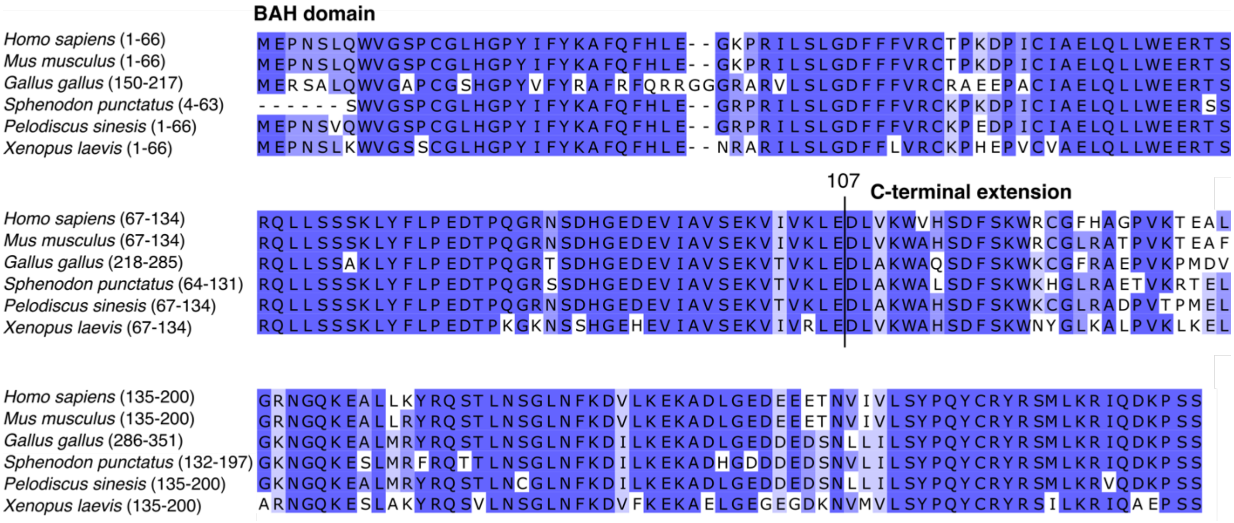

Figure S6: Structural alignment of the ARID5B BAH domain**;** h = human, b = bovine, m = murine

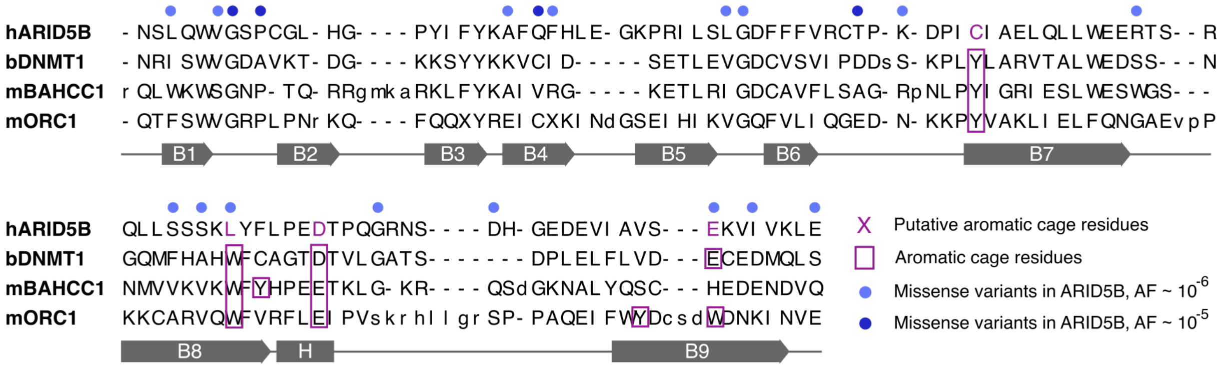

Supplement: Supplementary data 1 [file mmc1.pdf]
